# Emergency Physician Survey on Firearm Injury Prevention: Where Can We Improve?

**DOI:** 10.5811/westjem.2020.11.49283

**Published:** 2021-02-08

**Authors:** David A. Farcy, Nicole Doria, Lisa Moreno-Walton, Hannah Gordon, Jesus Sánchez, Luigi X. Cubeddu, Megan L. Ranney

**Affiliations:** * Mount Sinai Medical Center, Department of Emergency Medicine, Miami Beach, Florida; † Florida International University Herbert Wertheim College of Medicine, Department of Emergency Medicine & Critical Care, Miami Florida; ‡ Dalhousie University, School of Health and Human Performance, Halifax, Canada; § Louisiana State University Health Sciences Center, Department of Emergency Medicine, New Orleans, Louisiana; ¶ NOVA Southeastern University, Department of Socio Behavioral Sciences, COP, Davie, Florida; || NOVA Southeastern University, Department of Pharmaceutical Sciences, COP, Davie, Florida; # Brown University, Alpert Medical School, Department of Emergency Medicine, Providence, Rhode Island

## Abstract

**Introduction:**

Firearm injury and death is increasingly prevalent in the United States. Emergency physicians (EP) may have a unique role in firearm injury prevention. The aim of this study was to describe EPs’ beliefs, attitudes, practices, and barriers to identifying risk of and counseling on firearm injury prevention with patients. A secondary aim was assessment of perceived personal vulnerability to firearm injury while working in the emergency department (ED).

**Methods:**

We conducted a cross-sectional survey of a national convenience sample of EPs, using questions adapted from the American College of Surgeons’ Committee on Trauma 2017 survey of surgeons. Descriptive statistics and chi-square tests were calculated as appropriate.

**Results:**

A total of 1901 surveys were completed by EPs from across the United States. Among respondents, 42.9% had a firearm at home, and 56.0% had received firearm safety training. Although 51.4% of physicians in our sample were comfortable discussing firearm access with their high-risk patients, more than 70% agreed or strongly agreed that they wanted training on procedures to follow when they identify that a patient is at high risk of firearm injury. Respondents reported a variety of current practices regarding screening, counseling, and resource use for patients at high risk of firearm injury; the highest awareness and self-reported screening and counseling on firearm safety was with patients with suicidal ideation. Although 92.3% of EPs reported concerns about personal safety associated with firearms in the ED, 48.1% reported that there was either no protocol for dealing with a firearm in the ED, or if there was a protocol, they were not aware of it. Differences in demographics, knowledge, attitudes, and behavior were observed between respondents with a firearm in the home, and those without a firearm in the home.

**Conclusions:**

Among respondents to this national survey of a convenience sample of EPs, approximately 40% had a firearm at home. The majority reported wanting increased education and training to identify and counsel ED patients at high risk for firearm injury. Improved guidance on personal safety regarding firearms in the ED is also needed.

## INTRODUCTION

Firearm injury in the United States is a continuing epidemic.^1^,^2^ In 2017 alone, there were 39,773 firearm-related deaths: 23,854 suicides; 14,542 homicides; 486 resulting from unintentional discharge of a firearm; and 338 of undetermined origin.^3^ The rate of firearm death has increased 20% in the last five years.^4^ Although firearm injury statistics are unreliable, the best available data estimates that in the last five years there were more than twice as many nonfatal firearm injuries seen in emergency departments (ED).^5^ In 2018 and 2019, medical organizations joined together to assert the need for a public health approach to firearm injury, highlighting the need for research and describing ways in which the medical community could design and implement clinically-based firearm injury prevention initiatives.^6^,^7^

Physicians effectively risk stratify and counsel patients regarding preventive health including tobacco and alcohol cessation, correct use of infant car seats, the importance of wearing seatbelts and helmets, drowning prevention, and vaccinations.^8^–^10^ Evidence suggests that similar risk stratification and counseling discussions may be effective for preventing firearm injury and its consequences.^11^ Physicians can identify at-risk patients, provide factual information about firearm injury risk and, if needed, refer patients to resources that may reduce risk.^12^–^14^ Contrary to the myth that patients resent being counseled on firearm safety by their doctors, the literature shows that patients are receptive to discussing firearm injury prevention with physicians, as long as counseling is delivered in a respectful manner.^15^,^16^ While physicians who own firearms may be more likely to discuss firearm injury prevention with patients than those that don’t,^17^ in general, few physicians raise the subject with patients. This is true despite physicians in general believing they have the right to discuss firearm safety, and medical leadership groups and patients concurring and encouraging such discussions.^18^

There are approximately 150 million ED visits each year in the US.^3^ Emergency physicians (EP) are not only the first (and sometimes only) physicians to treat patients with firearm injuries, we also have a well-documented role in identification and implementation of injury prevention strategies in general.^19^ However, a recent study found that the charts of only 3% of patients presenting with suicidal ideation documented whether or not the patient had access to a firearm,^20^ and according to a small, non-scientific survey in 2016, few EPs discussed risk of firearm injury with victims of domestic violence, assault, or other high-risk categories.^21^ A survey of EPs in 22 states reported that although two-thirds of respondents had encountered a firearm in the ED, fewer than half felt at all confident in their ability to safely handle the situation.^22^ These missed opportunities may be related to the paucity of education on this topic in medical schools, or due to other unmeasured factors.^1^,^2^

Prior work conducted by the American College of Surgeons described attitudes, beliefs, and practices of US surgeons regarding firearms and firearm injury prevention, and was used to develop consensus recommendations on surgeons’ roles in firearm injury prevention.^23^ Given EPs’ critical role in injury prevention, a similar assessment of EPs is warranted. The aims of this study were to assess EPs’ knowledge, attitudes, and self-reported practice regarding firearm injury prevention, and to evaluate their perceived personal vulnerability to firearm injury in the workplace.

**Population Health Research Capsule**

What do we already know about this issue?

*Firearm injury and death is increasingly prevalent in the United States. Emergency physicians (EP) may have a unique role in firearm injury prevention*.

What was the research question?

*What are EPs’ beliefs, attitudes, practices, and barriers to identifying and counseling on firearm injury prevention*.

What was the major finding of the study?

*EP’s reported wanting increased education and training to identify and counsel ED patients at high risk for firearm injury*.

How does this improve population health?

*Education, training, protocols and open dialogue between EPs and patients may improve screening and counseling of at-risk patients - and, potentially, reduce incidence of firearm injury and death*.

## MATERIALS AND METHODS

A cross-sectional survey, adapted from the previously published American College of Surgeons’ Committee on Trauma (ACS-COT),^23^,^24^ was endorsed and distributed by the American Academy of Emergency Medicine (AAEM), the Resident Student Association (RSA/AAEM) and the US Council of Residency Directors in Emergency Medicine (CORD-EM). The questionnaire was sent via email and online newsletters to a convenience sample of ~6000 US resident and attending EPs using an online survey tool (SurveyMonkey, San Mateo, CA); the exact number of recipients is unknown, due to unknown overlap between survey lists. The survey opened on June 26, 2019 and remained open until August 31, 2019.

A consensus panel of experts in emergency medicine (EM) developed the survey items based on a 2017 survey from the ACS-COT.^23^,^24^ The final survey is available in Appendix 1. All authors reviewed, tested, and edited multiple iterations of the survey prior to approving the final version. No identifiers were incorporated to ensure the privacy of the respondents, and no individuals were identified in the analysis or written results. No incentives were awarded for completion of the survey.

Descriptive statistics were expressed as the number of observations, percentages, means ± standard error of the mean (SEM), and 95% confidence intervals (CI). For ease of analysis and presentation, some questions with four or five category outcomes were collapsed into a dichotomous variable (e.g., “always or almost always” vs “neutral, rarely, never”; or “strongly agree or agree” vs “neutral, disagree, or strongly disagree”). We conducted chi-square tests of association to examine the association between reporting owning a gun or having a firearm in the home, and an array of study participants’ characteristics, beliefs, knowledge, and attitudes. SPSS version 26 (IBM Corp., Armonk, New York) was used for statistical analysis.

The study was given exempt status by the Institutional Review Board at Mount Sinai Medical Center, Miami Beach, Florida. This research was conducted without grant funding or support from any public, commercial, or non-profit source.

## RESULTS

A total of 1901 respondents completed surveys, of whom 62.3% self-identified as men, 79.8% as White, and 64.3% as attending physicians (Table 1). All regions of the country were represented, with the highest proportion of responses (32.0%) from the Northeast. Three quarters (75.3%) of respondents identified their location of current practice or residency training site as a large city or a suburb near a large city. Most (86.1%) of the respondents were civilians without any military experience. Almost half (42.9%) reported having at least one firearm at home, of whom 84.8% personally owned the firearms (Table 1). More than half of participants (56.0%) had some prior training on firearm safety for personal use, more than half (57.1%) strongly agreed or agreed that personal ownership of firearms by private individuals in the US should be a constitutional right, and almost half (45.1%) strongly agreed or agreed that personal ownership of firearms protects personal liberty. Demographic differences were observed in who reported having a gun at home, with male (49.3%) and White (45.1%) respondents being more likely than women (30.3%), Hispanic (34.2%), and Black (22.8%) respondents, while rural (58.7%) and small town (51.9%) respondents reported being more likely to have a gun at home than respondents in large cities (38%) or suburbs (44.5%). Of respondents who considered gun ownership a constitutional right and a personal liberty, 81.0% and 85.9% reported having a gun at home. (Table 1).

**Figure 1:**
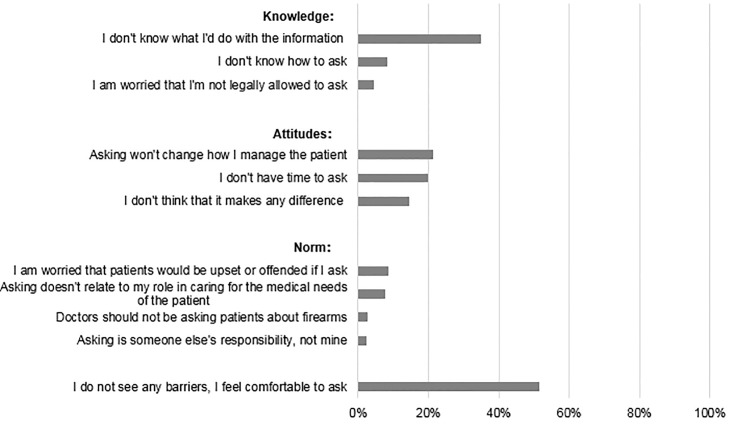
Participants were asked which of these are significant knowledge, attitudinal, and norm-related barriers to personally asking patients about firearm access. (Total n = 1,701.)

Regarding barriers to *asking* at-risk patients about firearms, most (51.4%) reported “no barriers to, or felt comfortable with, asking patients about firearm access” (Figure 1). Yet almost half (47.7%) reported lack of knowledge (e.g., “I don’t know what to do with the information”); more than half (55.8%) reported attitudinal barriers (e.g., “I don’t think it makes a difference”); and one-fifth (21.3%) reported negative attitudes and normative beliefs (e.g., “Asking is someone else’s responsibility, not mine”) about screening (Figure 1).

**Figure 2:**
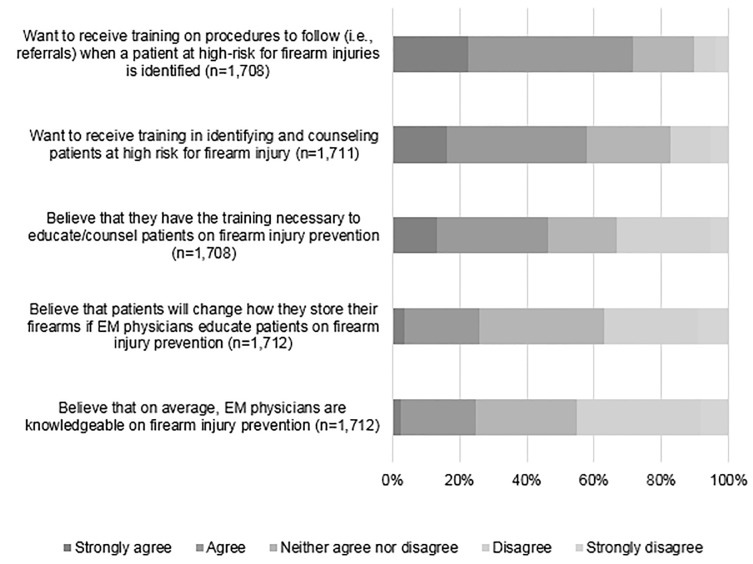
Participants agreement with the statements about training in firearm injury prevention (on a scale from strongly agree to strongly disagree). *EM*, emergency medicine.

Respondents had a wide variety of beliefs about *counseling* on firearm injury prevention. Only a quarter (25.7%) of respondents “strongly agreed” or “agreed” that patients would change how they store their firearms if physicians educated patients on firearm injury prevention. Almost half (46.1%) said that they personally had the training necessary to educate/counsel patients on firearm injury prevention. Nonetheless, nearly three-quarters (71.0%) wanted additional training in procedures to follow for patients at risk, and only a quarter (24.8%) “strongly agreed” or “agreed” that EPs in general are knowledgeable about firearm injury prevention (Figure 2).

**Figure 3:**
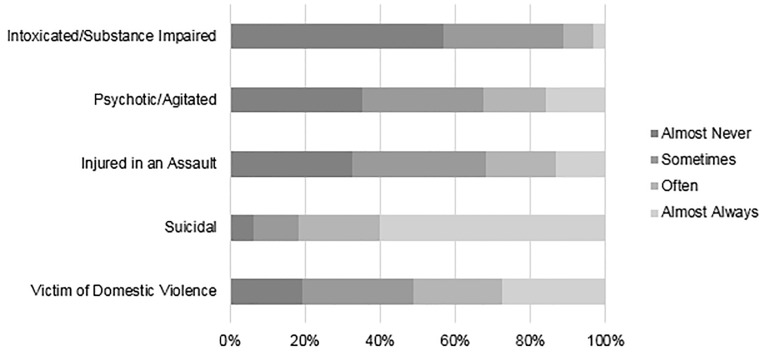
Frequency of asking a patient about firearm access in different scenarios. (Total n = 1,710)

**Figure 4:**
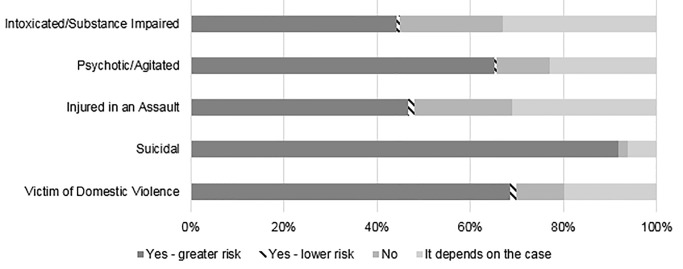
Knowledge of a patient’s firearm access changes assessment of risk of harm. (Total n=1,711)

**Figure 5:**
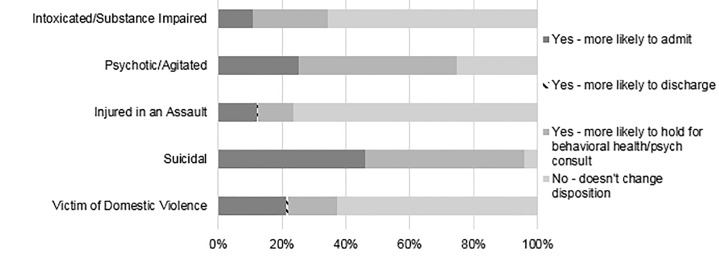
The proportion of participants that changed their assessment about a patient’s risk of future violence/self-harm if the patient was intoxicated/substance impaired (n = 1,704), psychotic/agitated (n = 1,704), injured in an assault (n = 1,703), suicidal (1,710), and/or a victim of domestic violence (1,707). Total participants who answered this question n = 1,711.

Self-reported frequency of asking patients about firearm access was dependent on the clinical scenario (Figure 3). Almost all (82.3%) EPs self-reported almost always or often asking a patient with suicidal ideation or suicide attempt (SI/SA) about firearm access, compared to 52.4% of cases where patients presented as victims of domestic violence, and lower rates for patients with psychosis or intoxication (11.7%). Knowing that a patient had access to a firearm would reportedly increase concern of future risk of violence or self-harm for 91.7 % for suicidal patients, vs only 46.6% of assault-injured patients (Figure 4). Knowing that a patient had access to a firearm would change an EP’s assessment of a patient only rarely, except for suicidal or psychotic patients (Figure 5). When asked about counseling, however, less than half (46.9%) of respondents reported “almost always” or “often” counseling suicidal patients and their families on lethal means.

Differences in responses were observed between respondents with a firearm in the home, and those without a firearm in the home. Although the majority (79%) of respondents with a firearm in the home believed that they had the training necessary to educate/counsel patients on firearm injury prevention, only 38.1% believed that other EPs were knowledgeable on firearm injury prevention. Of the EPs who strongly agreed that they wanted additional training in procedures to both identify and counsel patients at risk, only 26.4% and 22.9%, respectively, were gun owners (vs 73.6% and 77.1% non-gun owners; *P*<0.0001). Of EPs who strongly agreed that counseling would change how patients stored their firearms, only 34.4% were gun owners (vs 65.6% non-gun owners; *P*<0.0001). Compared to those without a firearm in the home, respondents with a firearm in the home were less likely to report that knowing a patient had firearm access changed their assessment about their risk of future violence/self-harm for a victim of domestic violence (30.6 vs 69.4%), a suicidal patient (38.2% vs 61.7%), an assault-injured patient (27.2% vs 72.8%), a psychotic/agitated patient (37.1% vs. 62.9%), or an intoxicated/substance impaired patient (27.9% vs 72.1%) (*P*<0.001). Yet respondents with firearms in the home more frequently reported asking about lethal means compared to non-gun owners (almost never asked: gun-owners 68%; non-gun owners: 32%; *P*<0.0001).

When asked, “How big a concern for you is your personal safety associated with firearms while you are working in the ED?,” only 7.7% responded “no concern at all”; 25.3% expressed “very great concern”; 36.8% expressed “moderate concern”; and 30.1% expressed “some concern.” Almost 40% (n = 654) of EPs responded that they did not know whether their ED had a procedure for securing patient firearms, and 9.8% said that no protocols existed. Respondents with a firearm in the home were less likely to report concern about their personal safety while working at the ED (very great concern: 35.9% gun owners vs 64.1% no gun owners, *P*<0.0001).

## DISCUSSION

To our knowledge, this study is the most comprehensive assessment to date of EPs’ attitudes, beliefs, and self-reported behaviors in relation to firearm injury prevention in the clinical setting. Despite respondents representing a convenience sample, the percent of respondents with a firearm in their home is similar to that reported in national surveys, and the geographic, gender, and racial/ethnic distribution of the respondents is similar to that in national data on emergeny medicine.^22^ Among this diverse sample of EPs, despite half reporting no barriers to asking high-risk patients about firearm access, numerous training needs were identified. The most notable findings were the disparities between reported knowledge, attitudes, and normative beliefs about the values of screening vs actual reported counseling of high-risk patients. There were stark disparities between what respondents said they did, and what others did. Differences in knowledge, attitudes, and beliefs about screening and counseling were also observed between firearm owners and non-owners.

Reassuringly, our survey identifies that neither knowledge nor normative beliefs are major barriers to firearm injury screening and counseling for high-risk patients. Most respondents reported knowing how to ask, and most reported that a positive finding would affect their judgment (but not necessarily their behavior) regarding evaluation of an at-risk patient. Only 8.6% reported being afraid to ask a patient about access to a firearm. This finding differs from other surveys of other physicians’ knowledge and attitudes, which reported low rates of knowledge about the incidence of firearm injury and discomfort with asking about firearms.^25^ This difference may reflect multiple medical societies’ educational efforts over the last half-decade emphasizing that patients are open to respectful, non-judgmental discussions of firearm injury risk.^26^,^27^

According to this survey, the two primary barriers to EPs’ effectively screening and counseling ED patients about firearm injury were not knowing how to respond to the information, and not thinking it will change management. Lack of resources, and skepticism about efficacy has been identified by others^22^,^25^–^28^ as common barriers to effective firearm injury prevention in the ED. Our findings, therefore, reinforce the importance of physician and patient self-training resources and handouts, In 2019, Pallin et al published a guide to when and how to intervene to reduce firearm injury.^11^ In response, multiple resources have been recently developed, including the following: 1) “What You Can Do” and “BulletPoints,” initiatives from University of California at Davis^29^; 2) “Gun Safety and Your Health” (available in both English and Spanish) from the American College of Surgeons^30^; 3) Guides to home firearm safety and pediatric counseling from the Firearm Safety Among Children and Teens (FACTS) Consortium^31^; 4) safe storage resources from the Colorado Firearm Safety Coalition^32^; and 5) a compendium of resources from the American Foundation for Firearm Injury Reduction in Medicine (AFFIRM), a non-partisan network of health professionals dedicated to changing the conversation about firearm injury prevention.^33^ Emergency departments interested in decreasing barriers to screening and intervention could review and share these well-developed resources.

In line with national surveys, having a firearm in the home was more common among White men, those practicing in rural areas and small cities/towns, and those who believe that gun ownership is a constitutional right, a personal liberty, and a self-protection.^34^ Those EPs with a firearm in the home were more likely to ask patients about lethal means, reported less concerns about their safety while working at the ED, were less interested in wanting additional training to identify patients at risk, and were less likely to agree that counseling would change how patients stored their firearms. Additionally, EPs with a firearm in the home were less likely than those without a firearm in the home to report insufficient knowledge about how to ask. These findings concord with our and others’ work showing that firearm owners can help lead evidence-based interventions to reduce firearm injury risk.^22^,^28^,^35^–^37^ Future educational programs should make an effort to highlight the voices, expertise, and experience of firearm-owning EPs.^37^,^38^ Nonetheless, deficits in knowledge were identified among this group, including lack of belief in the value of screening or counseling for patients who were at risk of non-suicide-related firearm injury.

The findings also suggest, unfortunately, that simple knowledge alone is unlikely to change behavior. For example, despite most participants reporting that screening is important and would change their behavior, and most respondents saying that they personally were comfortable with firearm counseling, almost all said that *other* EPs were not comfortable screening or counseling at-risk patients, and most requested at least some additional training for themselves. Similarly, despite most participants reporting that they “always or almost always” screen suicidal patients for firearm access (much higher than previous literature has reported),^20^,^26^,^39^ and most participants reporting that this knowledge would change their disposition decision for suicidal patients, less than half report delivering lethal means counseling. These incongruities may reflect social desirability bias (e.g., it may be easier for respondents to admit that others were unsure of what to do or how to do it, compared to admitting it about themselves). Others’ work has studied physicians’ actual behavior, using both electronic health records and self-report, and has similarly found that physicians screen far less often than self-report.^11^,^26^,^39^,^40^ Even if a large percentage of subjects in this study are asking patients with suicidal ideation about firearm access, competent counseling should be part of the discussion.^20^

The contradictions in responses may reflect a key lesson of behavior change theory^41^,^42^ and dissemination and implementation research: Attention must be paid to not just internal factors, but also healthcare and societal structures that influence change.^42^ For example, Runyan et al have suggested that having departmental written protocols for lethal means counseling has been associated with a higher rate of counseling for all suicidal patients, and that developing such standard protocols across the country might increase lethal mean counseling.^40^ Betz et al have developed physician-independent, web-based, lethal means counseling resources, with high acceptability and feasibility.^43^ Development and dissemination of similar resources that reduce physician burden and address physician-independent barriers may be necessary.

Finally, our data confirm that EPs were significantly concerned about their safety associated with firearms while working in the ED, with a quarter expressing “very great” and more than a third expressing “moderate concern” about their personal safety. This concern is exacerbated by both a lack of policy regarding firearm handling, and a lack of knowledge of any existing policies; the majority of respondents reported that they are concerned for their own safety, yet a third had no idea whether a policy existed. This finding could potentially be explained by several factors including physicians’ attitude toward the subject, professional priorities, or a lack of education or communication on the topic from ED leadership. In a survey conducted by Ketterer et al, 20% of attending and 25% of resident physicians reported encountering firearms in the ED or its immediate surroundings. Attending physicians, however, had more knowledge of hospital policy regarding handling and management of the firearm once it was discovered in a patient’s possession, as compared to residents.^22^–^28^ In another study Ketterer et al reports that “up to 25% of trauma patients brought to the emergency department (ED) have been found to carry weapons.”^28^ Overall, more research is needed to address safety in the ED and the handling of firearms when they are brought into the department; further collaborative work is needed.^24^,^45^

The American College of Surgeons’ Committee on Trauma^23^ published results from a similar survey of surgeons in 2016, with the primary objectives of identifying advocacy initiatives and efforts related to firearm safety. Our respondents were similar to ACS’ in demographics, percent firearm ownership, percent with gun safety training, and percent with a military background; the one major difference is that our EM survey included resident physicians, while the ACS survey did not. ACS found that the vast majority of respondents believed that healthcare professionals should be allowed to counsel patients on firearm safety and injury prevention, with 88% setting injury prevention as a high priority and 94% responding that federal funding should be allocated for firearm safety and injury prevention research.^23^ Our study, conducted two years later after extensive educational work by both ACS and EM professional societies,^7^,^45^ assumed that healthcare professionals have the duty to discuss firearm safety and injury prevention with at-risk patients, and sought instead to determine how often these conversations were taking place (< 50% of encounters with suicidal patients), how comfortable physicians were in having these conversations (51.4%), and what percentage of physicians felt the need for further training to effectively engage patients in these conversations (>70%).

The overarching theme of our organizations, institutions and collaborations is to explore shared goals among healthcare professionals, public health researchers, educators, advocates, firearm owners, gun shops,^46^ and law enforcement officials who are collectively committed to working toward suicide prevention and firearm safety.^32^ Our study supports the need for increased training and protocols regarding firearm counseling, handling, and medical record documentation. Physicians are aware of the lack of training and are open to learning the necessary skills to save lives through education and prevention of firearm injuries. Further research is needed on the efficacy of current training and available resources.

## LIMITATIONS

Selection bias is always present when a survey is sent to one or more large organizations by email; it is likely that respondents have stronger feelings or opinions about the survey topic. Another limitation associated with survey studies is the potential for over- or under-reporting of results due to inaccuracies attributable to social desirability or recall biases. However, social desirability bias has been shown to be less likely to occur with online surveys, such as ours, where no personal identifiers are involved and responses are more accurate than those obtained from face-to-face or telephone surveys.^47^,^48^ This study is subject to a geographic bias, since most respondents were from the East coast of the US, although geographic bias is far more likely to impact results when surveys are done in various countries whose socioeconomic, religious, and political climates may vary considerably.

## CONCLUSION

Emergency physicians, whether firearm owners or not, believe in the importance of screening and counseling to reduce risk of firearm injury among at-risk patients. Nonetheless, further training, resources, and innovative interventions are needed to aid EPs in accurate identification and management of these high-risk patients. Additional resources are also needed to increase knowledge about personal safety from firearm injury in the ED.

## Supplementary Information



## Figures and Tables

**Table 1:** Demographics and characteristics of survey participants (N=1,901).

Characteristics	Total % (N)
Gender (n = 1901)	
Male	62.3 (1,185)
Female	36.0 (684)
Rather not answer	1.5 (29)
Other	0.2 (3)
Race and Ethnicity (n = 1893)	
White	79.8 (1,511)
Asian or Asian American	9.2 (174)
Hispanic/Latino	6.2 (118)
Other	3.8 (72)
Black or African American	3.7 (69)
Middle East/North Africa	1.8 (34)
Native American or Alaska Native	0.7 (13)
Level of Training in Emergency Medicine (n = 1898)	
Attending 1–5 year out of residency	23.1 (439)
Attending more than 16 years out of residency	15.9 (301)
Attending 6–10 years out of residency	15.5 (294)
Resident PGY 1	13.3 (252)
Attending 11–15 years out of residency	9.9 (187)
Resident PGY 3	9.9 (187)
Resident PGY 2	7.9 (150)
Other	2.2 (42)
Resident PGY 4	2.2 (41)
Resident PGY 5	0.3 (5)
Region of Practice (n = 1825)	
Northeast	32.0 (584)
Southeast	24.0 (438)
Midwest	16.0 (292)
Southwest	14.0 (256)
West	14.0 (255)
Location of Current Practice or Training (n = 1897)	
Large city	54.9 (1,042)
Suburb near a large city	20.4 (386)
Small city or town	19.6 (371)
Rural area	3.4 (65)
Other	0.9 (17)
Not currently in a clinical practice	0.8 (16)
Has military experience (previous or active)	13.9 (263)
No military experience (previous or active)	86.1 (1,635)
Has training on firearms safety for personal purposes	56.0 (1,063)
No training on firearms safety for personal purposes	44.0 (835)
Has firearms stored in home (even if not owner)	42.9 (806)
Personal owner of firearm stored in home	84.9 (656)
No firearms stored in home (even if not owner)	57.1 (1,074)

Notes: Total number of participants in study is N = 1 901. Participants could skip questions, which is why different questions have different n.

*PGY*, postgraduate year.

## References

[1] Butkus R, Doherty R, Bornstein SS (2018). Reducing firearm injuries and deaths in the United States: a position paper from the American College of Physicians. Ann Intern Med.

[2] Weinberger SE, Hoyt DB, Lawrence HC (2015). Firearm-related injury and death in the United States: a call to action from 8 health professional organizations and the American Bar Association. Ann Intern Med.

[3] Centers for Disease Control and Prevention (2012). About Underlying Cause of Death 1999–2010.

[4] Centers for Disease Control and Prevention, National Center for Injury Prevention and Control WISQARS (Web-based Injury Statistics Query and Reporting System).

[5] Services H, Control D, Prevention I (2011). Firearm Injury Surveillance Study, 1993–2008.

[6] Bulger EM, Kuhls DA, Campbell BT (2019). Proceedings from the Medical Summit on Firearm Injury Prevention: a public health approach to reduce death and disability in the US. J Am Coll Surg.

[7] Ranney ML, Betz ME, Dark C (2019). #ThisIsOurLane — Firearm Safety as Health Care’s Highway. N Engl J Med.

[8] Schlaff AL (2013). Behavior change in America: Public health, medicine, and individual counseling. Virtual Mentor.

[9] McGinnis JM, Hamburg MA (1988). Opportunities for health promotion and disease prevention in the clinical setting. West J Med.

[10] Theurer WM, Bhavsar AK (2013). Prevention of unintentional childhood injury. Am Fam Physician.

[11] Pallin R, Spitzer SA, Ranney ML (2019). Preventing firearm-related death and injury. Ann Intern Med.

[12] McLean RM, Harris P, Cullen J (2019). Firearm-related injury and death in the United States: A call to action from the nation’s leading physician and public health professional organizations. Ann Intern Med.

[13] Damari ND, Ahluwalia KS, Viera AJ (2018). Continuing medical education and firearm violence counseling. AMA J Ethics.

[14] Jones N, Nguyen J, Strand NK (2018). What should be the scope of physicians’ roles in responding to gun violence?. AMA J Ethics.

[15] Betz ME, Azrael D, Barber C (2016). Public opinion regarding whether speaking with patients about firearms is appropriate: results of a national survey. Ann Intern Med.

[16] Boge LA, Dos Santos C, Burkholder JD (2019). Patients’ perceptions of the role of physicians in questioning and educating in firearms safety: post-FOPA repeal era. South Med J.

[17] Becher EC, Cassel CK, Nelson EA (2000). Physician firearm ownership as a predictor of firearm injury prevention practice. Am J Public Health.

[18] Butkus R, Weissman A (2014). Internists’ attitudes toward prevention of firearm injury. Ann Intern Med.

[19] American College of Emergency Physicians (ACEP) (2011). Firearm injury prevention. Policy statement. Ann Emerg Med.

[20] Naganathan S, Mueller KL (2019). Physician documentation of access to firearms in suicidal patients in the emergency department. West J Emerg Med.

[21] Ranney ML, Barsotti C (2016). Opinion: Firearm injury prevention is more than pro/con debate.

[22] Ketterer AR, Poland S, Ray K (2020). Emergency providers’ familiarity with firearms: a national survey. Acad Emerg Med.

[23] Kuhls DA, Campbell BT, Burke PA (2017). Survey of American College of Surgeons Committee on trauma members on firearm injury: Consensus and opportunities. J Trauma Acute Care Surg.

[24] Ranney ML, Fletcher J, Alter H (2017). A consensus-driven agenda for emergency medicine firearm injury prevention research. Ann Emerg Med.

[25] Roszko PJD, Ameli J, Carter PM (2016). Clinician attitudes, screening practices, and interventions to reduce firearm-related injury. Epidemiol Rev.

[26] Betz ME, Miller M, Barber C (2016). Lethal means access and assessment among suicidal emergency department patients. Depress Anxiety.

[27] Betz ME, Wintemute GJ (2015). Physician counseling on firearm safety: a new kind of cultural competence. JAMA.

[28] Ketterer AR, Ray K, Grossestreuer A (2019). Emergency physicians’ familiarity with the safe handling of firearms. West J Emerg Med.

[29] UC Davis Health (2020). What You Can Do Home.

[30] American College of Surgeons (2020). Firearm Injury Prevention Activities.

[31] (2018). Cleveland Metropolitan School District. Facts/Home.

[32] Colorado Firearm Safety Coalition (2019). Gun Storage Map.

[33] AFFIRM Research What We Do.

[34] Saad L (2019). What percentage of Americans own guns?.

[35] Pallin R, Spitzer SA, Ranney ML, Betz ME, Wintemute GJ (2019). Preventing firearm-related death and injury. Ann Intern Med.

[36] Pallin R, Siry B, Azrael D (2019). “Hey, let me hold your guns for a while”: a qualitative study of messaging for firearm suicide prevention. Behav Sci Law.

[37] Betz ME, Bebarta VS, DeWispelaere W (2019). Emergency physicians and firearms: effects of hands-on training. Ann Emerg Med.

[38] Ketterer AR, Ray K, Grossestreuer A (2019). Emergency physicians’ familiarity with the safe handling of firearms. West J Emerg Med.

[39] Betz ME, Kautzman M, Segal DL (2018). Frequency of lethal means assessment among emergency department patients with a positive suicide risk screen. Psychiatry Res.

[40] Runyan CW, Brooks-Russell A, Tung G (2018). Hospital emergency department lethal means counseling for suicidal patients. Am J Prev Med.

[41] Ajzen I (1991). The theory of planned behavior. Organ Behav Hum Decis Process.

[42] Damschroder LJ, Aron DC, Keith RE (2009). Fostering implementation of health services research findings into practice: A consolidated framework for advancing implementation science. Implement Sci.

[43] Betz ME, Knoepke CE, Siry B (2018). “Lock to Live”: Development of a firearm storage decision aid to enhance lethal means counselling and prevent suicide. Inj Prev.

[44] Talley CL, Campbell BT, Jenkins DH (2019). Recommendations from the American College of Surgeons Committee on Trauma’s Firearm Strategy Team (FAST) Workgroup: Chicago Consensus I. J Am Coll Surg.

[45] Bulger EM, Kuhls DA, Campbell BT (2019). Proceedings from the Medical Summit on Firearm Injury Prevention: a public health approach to reduce death and disability in the US. J Am Coll Surg.

[46] Rabin RC (2020). ‘How did we not know?’ Gun owners confront a suicide epidemic.

[47] Wertz J, Azrael D, Hemenway D (2018). Differences between new and long-standing US gun owners: results from a national survey. Am J Public Health.

[48] Kreuter F, Presser S, Tourangeau R (2008). Social desirability bias in CATI, IVR, and web surveys: the effects of mode and question sensitivity.

