# Supplementary material for: Emergency Physician Survey on Firearm Injury Prevention: Where Can We Improve?
Source: West J Emerg Med. 2021 Feb 8;22(2):257–65. doi: 10.5811/westjem.2020.11.49283 (PMC7972360; doi:10.5811/westjem.2020.11.49283)
Supplement: Supplementary file 1 [file wjem-22-257-s001.docx]

**Appendix 1. Emergency Medicine Physicians Firearm Injury Prevention Survey**

Thank you for taking the time to participate in this study! The purpose of the survey is to better understand the views of U.S. Emergency Medicine resident and attending physicians on topics related to firearm injury prevention. This study has been approved through the IRB at Mount Sinai Medical Center, Miami Beach, Florida. The final report will draw upon aggregated responses. All individual answers are completely anonymous, and no personal information is collected. The survey takes approximately 5 minutes to complete. We appreciate your time! You may receive this study from different Emergency Medicine groups but please only answer survey one time.

1. What is your gender?

Female

Male

Other

Rather not answer

2. What is your race and ethnicity (mark all that apply)?

Hispanic/Latino

White or Caucasian

Black or African American

Asian or Asian American

Native American or Alaska Native

Middle East/North Africa

Other

3. What best describes your level of training in Emergency Medicine?

Resident PGY 1

Resident PGY 2

Resident PGY 3

Resident PGY 4

Resident PGY 5

Attending 1-5 year out of residency

Attending 6-10 years out of residency

Attending 11-15 years out of residency

Attending more than 16 years out of residency

Other (please specify):

4. In which state do you practice Emergency Medicine?

5. Which of the following best describes the location of your current practice or training location?

Large city

Suburb near a large city

Small city or town

Rural area

Other

I am not currently in a clinical practice

6. Which of the following best describes your military experience?

No military service record; civilian

Previous OR current Active Duty, Reserves, and/or National Guard

7. Have you ever received any training on firearms safety for personal purposes?

Yes

No

8. Which of the following firearms safety training(s) for personal purposes have you received? Select all that apply:

Summer Camp

Hunter safety course

Military

Concealed carry course

Boy Scouts

Firing Range

Other

9. Are there currently any firearms stored in your home, even if you are not the owner?

Yes

No

10. Who owns the firearms stored in your home (please check all that apply)?

I do

Another member of the household

Someone who does not live in my household

Other (please specify):

11. Which of the following firearms or accessories are currently stored in your home (please check all that apply)?

Long gun(s) (rifle or shotgun)

Long gun(s) high capacity (>10round) magazine fed, semi-automatic rifle (AKAAR-15 equivalent)

Long gun(s) high capacity magazine fed, automatic rifle or other NFA Class III regulated firearm

Bump stock or trigger actuator

Handgun(s) (pistols, revolvers, Derringer)

12. What is the purpose of the firearms that are stored in your household (please check all that apply)?

Target shooting

Hunting

Self-defense/Protection

Collecting

Competition

Other (please specify):

13. Which of the following describes how firearms are stored in your home (please check all that apply)? For example, if you have two guns, and one is locked in a gun safe and the other is unlocked, please check both of these answers.

Locked in a gun safe

Locked with a trigger lock

Unlocked

14. I believe that personal ownership of firearms by private individuals in the United States should be a constitutional right.

Strongly agree

Agree

Neither agree nor disagree

Disagree

Strongly disagree

15. Do you believe that personal ownership of guns by private individuals in the United States protects personal liberty?

Strongly agree

Agree

Neither agree nor disagree

Disagree

Strongly disagree

16. How often do you ask about firearm access if a patient (check all that apply)...

Is a victim of domestic violence?

Is suicidal?

Was injured in an assault?

Is psychotic/agitated?

Is intoxicated/substance impaired?

Almost never

Sometimes

Often

Almost always

17. Does knowing that a patient has firearm access change your assessment about their risk of future violence/self-harm for (check all that apply):

A victim of domestic violence

A suicidal patient

An assault-injured patient

A psychotic/agitated patient

An intoxicated/substance impaired patient

Yes-I think they are greater risk

Yes-I think they are lower risk

No, it doesn't change my assessment

It depends on the case

18. Does knowing that a patient has firearm access change your disposition decision for (check all that apply):

A victim of domestic violence

A suicidal patient

An assault-injured patient

A psychotic/agitated patient

An intoxicated/substance impaired patient

Yes- I am more likely to admit them

Yes- I am more likely to discharge them

Yes- I am more likely to hold them for a behavioral health/psych consult

No, it doesn't change my disposition

19. How often do you counsel suicidal patients and their families about decreasing access to "lethal means" (eg: locking up dangerous medicines; locking up guns or storing them with a family member)?

Almost never

Sometimes

Often

Almost always

20. Which of these are significant barriers to you, personally, asking your patients in your ED about firearm access (please check all that apply)?

I don't know how to ask.

I don't have time to ask.

I don't know what I'd do with the information. (eg, what resources or counseling to provide)

I don't think that it makes any difference. (I can't change the outcome)

I am worried that patients would be upset or offended if I ask.

I am worried that I'm not legally allowed to ask.

Asking won't change how I manage the patient.

Asking is someone else's responsibility, not mine.

Doctors should not be asking patients about firearms.

Asking doesn't relate to my role in caring for the medical needs of the patient.

I do not see any barriers, I feel comfortable to ask.

21. Does your Emergency Department have a procedure on how to secure patients' firearms while they are in the facility?

Yes

No

I don't know

22. How big a concern for you is your personal safety associated with firearms while you are working in the

Emergency Department?

Very great concern

Moderate concern

Small concern

Not a concern at all

23. I believe that patients will change how they store their firearms if EM physicians educate patients on

firearm injury prevention.

Strongly agree

Agree

Neither agree nor disagree

Disagree

Strongly disagree

24. I believe that on average, EM physicians are knowledgeable on firearm injury prevention.

Strongly agree

Agree

Neither agree nor disagree

Disagree

Strongly disagree

25. I believe that I have the training necessary to educate/counsel patients on firearm injury prevention.

Strongly agree

Agree

Neither agree nor disagree

Disagree

Strongly disagree

26. I want to receive training in identifying and counseling patients at high risk for firearm injury.

Strongly agree

Agree

Neither agree nor disagree

Disagree

Strongly disagree

27. I want to receive training on procedures to follow (i.e., referrals) when a patient at high-risk for firearm injuries is identified.

Strongly agree

Agree

Neither agree nor disagree

Disagree

Strongly disagree

28. Florida physicians only: I believe that a mandatory CME course on firearm injury prevention, such as the HIV course and the domestic violence course, should be implemented.

Strongly agree

Agree

Neither agree nor disagree

Disagree

Strongly disagree

29. I believe that training in emergency medicine-based firearm injury prevention should include (please check all that apply):

Epidemiology of firearm accidents

Identification of patients at risk

Education and counseling of patients at risk

Procedures to follow for patients at risk

Training in proper handling of firearms

Other (please specify):
